# Protein-enriched outer membrane vesicles as a native platform for outer membrane protein studies

**DOI:** 10.1038/s42003-018-0027-5

**Published:** 2018-04-05

**Authors:** Johannes Thoma, Selen Manioglu, David Kalbermatter, Patrick D. Bosshart, Dimitrios Fotiadis, Daniel J. Müller

**Affiliations:** 1 Department of Biosystems Science and Engineering, Eidgenössische Technische Hochschule (ETH) Zürich, Mattenstrasse 26, Basel, 4058 Switzerland; 2 Institute of Biochemistry and Molecular Medicine, University of Bern, Bühlstrasse 28, Bern, 3012 Switzerland

## Abstract

Most studies characterizing the folding, structure, and function of membrane proteins rely on solubilized or reconstituted samples. Whereas solubilized membrane proteins lack the functionally important lipid membrane, reconstitution embeds them into artificial lipid bilayers, which lack characteristic features of cellular membranes including lipid diversity, composition and asymmetry. Here, we utilize outer membrane vesicles (OMVs) released from *Escherichia coli* to study outer membrane proteins (Omps) in the native membrane environment. Enriched in the native membrane of the OMV we characterize the assembly, folding, and structure of OmpG, FhuA, Tsx, and BamA. Comparing Omps in OMVs to those reconstituted into artificial lipid membranes, we observe different unfolding pathways for some Omps. This observation highlights the importance of the native membrane environment to maintain the native structure and function relationship of Omps. Our fast and easy approach paves the way for functional and structural studies of Omps in the native membrane.

## Abstract (web summary)

Johannes Thoma et al. overexpress outer membrane proteins (Omps) in *Escherichia coli* and collect the expelled outer membrane vesicles (OMVs) to study Omp assembly, folding and structure. They find that Omps in OMVs show different unfolding pathways compared to Omps reconstituted in artificial lipid membranes.

## Introduction

Cellular membranes shape and protect cellular compartments. They are mainly formed by lipids and membrane proteins, which provide essential cellular functions including adhesion, energy conversion, molecular transport, signaling, and communication. Being involved in key cellular functions, membrane proteins continuously attract attention in biology, medicine, and pharmacology^1–3^. To maintain their structural integrity and functionality, membrane proteins require the unique environment of an amphiphilic lipid bilayer. Thereby the specific lipid composition of a cellular membrane can modulate the functional state of the protein^4,5^. In eukaryotic membranes so-called lipid rafts dynamically reassemble raft-specific lipids and membrane proteins to adapt the functional state of the proteins to the cellular state^6^. For example the functional state of G-protein-coupled receptors like rhodopsin or the beta2-adrenergic receptor is correlated to the presence of cholesterol^7^. This lipid-dependent behavior is not restricted to eukaryotic membrane proteins. For example, the *E. coli* lactose permease LacY is sensitive to the phosphatidylethanolamine content of the membrane and adopts a non-functional conformation in phosphatidylethanolamine depleted membranes^8,9^. This sensitivity of the fold of membrane proteins to the phospholipid composition of the cytoplasmic membrane is observed frequently^10^. Another intriguing example is the insertion and folding of membrane proteins, which depends on the lipid composition and asymmetry of cellular membranes^11–14^.

The characterization of the folding, structure, and function of membrane proteins using biochemical and biophysical techniques requires proteins at sufficient purity and often at relatively high concentration. Exceptionally, native membranes fulfill these requirements, including the purple membrane from *Halobacterium salinarum*, which contains the membrane protein bacteriorhodopsin packed in two-dimensional lattices^15,16^ or disc membranes from rod outer segments of the vertebrate eye, which contain rows of rhodopsin dimers packed at high density^17–19^. However, the majority of functional and structural studies of membrane proteins rely on samples that have been extracted from native membranes by solubilization and reconstituted into artificial lipid bilayers. Reconstitution generally follows a three-step protocol: The membrane protein of interest is first solubilized using detergents, then purified, and finally reconstituted into bilayers assembled from synthetic or natural lipids^20^. Each of these steps needs to be specifically adapted for a particular membrane protein and it can take several months to years to establish an adequate reconstitution protocol. Although membrane proteins reconstituted into artificial lipid bilayers are frequently used for biophysical and biochemical studies, the bilayers can only roughly mimic the native environment of the much more complex cellular membrane. This is because essential properties such as the specific lipid composition and the unique lipid asymmetry of the two leaflets of the bilayer are lost during reconstitution. Furthermore, most proteins are oriented randomly in the bilayer after reconstitution. In an attempt to overcome these problems, we here introduce a fast and easy approach to prepare outer membrane proteins (Omps) in native membranes.

Outer membranes of Gram-negative bacteria are composed of two highly asymmetric membrane leaflets. Whereas the inner leaflet is composed of regular phospholipids, the outer leaflet consists almost exclusively of lipopolysaccharides (LPS)^21,22^. In a naturally occurring phenomenon, Gram-negative bacteria release outer membrane vesicles (OMVs) by bulging the outer membrane outwards. These OMVs have the membrane composition of the parental outer membrane^23–25^ with Omps forming various channels involved in the translocation of molecular compounds including several classes of antibiotics^26,27^. While the mechanism of OMV formation and their biological roles are still under investigation, their technological potential has been recognized. Exhibiting an outer surface, which is almost identical to that of bacteria but not containing the infectious organism, OMVs are promising vaccines^28^. In addition, the recent development of so-called designer-OMVs with specifically tuned surface properties opens an avenue for the usage of OMVs as biotechnological tools^29^. Here, we introduce OMVs as platform for the structural and functional characterization of Omps in their native membrane. To address a particular Omp we enriched it in the bacterial outer membrane and collected OMVs containing the Omp at high density and purity of up to ≈90%. Using complementary biochemical and biophysical methods, we characterize the folding, structure, and assembly of Omps within OMVs, thus demonstrating the general applicability of these samples for biological, pharmaceutical, and medical studies of membrane proteins under native-like conditions.

## Results

### Preparing OMVs carrying high amounts of selected proteins

**Fig. 1:**
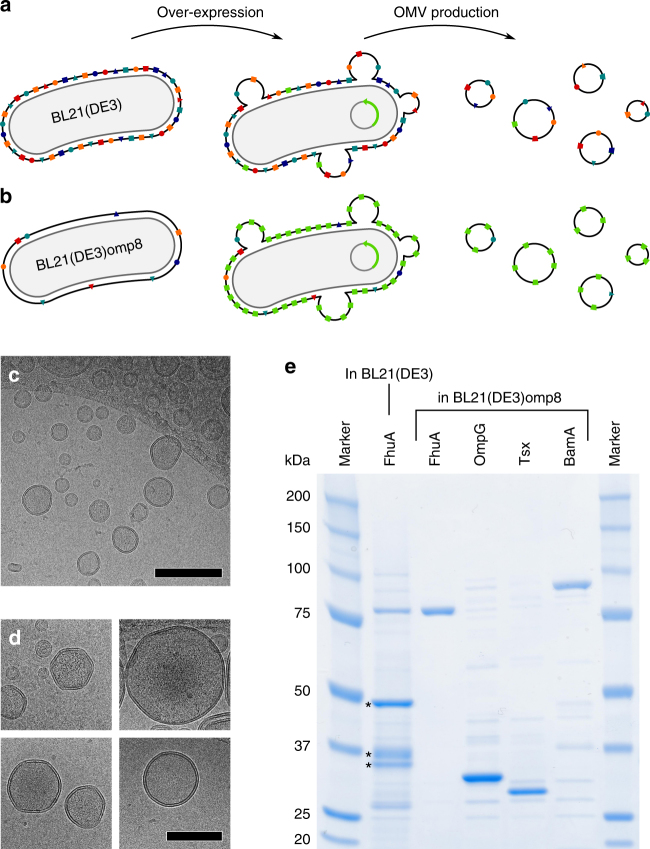
Preparation and analysis of outer membrane protein (Omp)-enriched outer membrane vesicles (OMVs). **a**, **b** To characterize the folding and structure of outer membrane proteins (OMPs) in their native environment we prepared outer membrane vesicles (OMVs) from two different *E. coli* strains. **a** The widely used *E. coli* expression strain BL21(DE3) contains large quantities of four major porins, namely OmpA, OmpF, OmpC, and LamB, which leaves limited space for the overexpression of additional OMPs. **b** Using strain BL21(DE3)omp8, which lacks the four major porins, allows overexpressing high amounts of selected OMPs. **c**, **d** Collected OMVs ranged from 50 to 250 nm in diameter, as observed by cryo-transmission electron microscopy. Shown are OMVs enriched in FhuA, for all other OMVs see Supplementary Fig. 2. **d** The majority of OMVs appeared unilamellar and intact. Scale bars, 250 nm (**b**) and 100 nm (**d**). **e** SDS-PAGE of OMVs collected after overexpression of FhuA in *E. coli* BL21(DE3), as well as OMVs collected after overexpression of FhuA, OmpG, Tsx, and BamA, respectively, in *E. coli* BL21(DE3)omp8. Whereas OMVs from BL21(DE3) cells show large quantities of native porins (marked by asterisks), OMVs from BL21(DE3)omp8 cells show a clear band of the overexpressed protein

To investigate Omps in the native outer membrane we searched for ways to overexpress Omps in *E. coli* and to purify the OMVs released by the bacteria (Fig. 1a and Methods). To this end, we used *E. coli* expression strains BL21(DE3) and BL21(DE3)omp8. The latter strain lacks the four major Omps, i.e., OmpA, OmpF, OmpC, and LamB, and has been shown to yield much higher levels of overexpressed Omps than strains having unaltered outer membranes^30^. To produce enriched OMVs, bacteria were grown to an optical density (OD_600_) of 0.4. Then, the expression of the Omp of interest was induced and maintained until cells reached the late exponential growth phase. After removal of the bacteria from the growth media by centrifugation and ultrafiltration, the OMVs released during bacterial growth were isolated from the supernatant by ultracentrifugation (Methods and Supplementary Fig. 1). Finally, the OMVs were washed with buffer and recollected by ultracentrifugation. The purification of OMVs showed prominent differences in the amounts of vesicles released from the different *E. coli* expression strains. To quantify these differences, we used the total protein content of the OMVs as a measure for the vesicle yield. Whereas OMV preparations from 250 mL BL21(DE3)omp8 liquid culture yielded ≈500 µg total protein, OMV preparations from 250 mL BL21(DE3) liquid culture yielded only ≈50 µg total protein. *E. coli* BL21(DE3)omp8 thus released ≈10-fold more vesicles than *E. coli* BL21(DE3). This difference is likely due to deletions of the *ompA* and *ompC* genes in the BL21(DE3)omp8 strain, which have been shown to increase vesiculation compared to *E. coli* strains having unaltered outer membranes^31,32^.

Cryo-transmission electron microscopy (cryo-TEM) of purified OMVs showed unilamellar intact vesicles, the diameters of which ranged from ≈50 to 250 nm (Fig. 1c, d and Supplementary Fig. 2). However, several vesicles were not spherical in shape but contained flattened regions, which reflected the densely packed assembly of Omps in the native outer membrane of Gram-negative bacteria^33,34^. We observed similar morphologies among the OMVs collected from either bacterial strain and independently of which particular Omp gene the strain overexpressed (Supplementary Fig. 2). Next, we analyzed the OMVs by SDS-PAGE (Fig. 1e). OMVs produced by *E. coli* BL21(DE3), which had been induced to overexpress FhuA, showed strong bands migrating at molecular weights between 25 and 50 kDa. These bands indicated the presence of the four major Omps OmpA, OmpF, OmpC, and LamB with molecular weights of 35.2, 37.1, 38.3, and 47.3 kDa, respectively^30^. At ≈80 kDa a weak band indicated minor amounts of FhuA^35^. This result confirmed that the unaltered outer membrane is highly enriched in major Omps, which leaves little potential for the insertion of overexpressed Omps^30^. In contrast, OMVs prepared from *E. coli* BL21(DE3)omp8 overexpressing either FhuA, OmpG, Tsx, or BamA showed single prominent bands (Fig. 1e). The prominent bands migrated at the expected molecular weight of 79.9 kDa for FhuA, 34.3 kDa for OmpG, 33.7 kDa for Tsx and 88.3 kDa for BamA. A faint background pattern of bands migrating at various heights between 20 and 100 kDa was present in all OMV samples. Analysis of the band intensities indicated that the fraction of overexpressed Omps ranged from ≈50 to ≈90% in OMVs released from *E. coli* BL21(DE3)omp8 (Supplementary Fig. 3). In contrast, OMVs collected from *E. coli* BL21(DE3) overexpressing FhuA contained only a fraction of ≈7% FhuA.

To analyze the Omp composition, the OMVs collected from overexpressing BL21(DE3)omp8 cultures were subjected to mass spectrometry (Supplementary Fig. 3 and Table 1). For every OMV preparation, the presence of the overexpressed Omp was confirmed. The mass spectrometry analysis also provided insight into the other proteins the OMVs contained. This protein background, which was similar for all OMV preparations, consisted mainly of soluble periplasmic proteins and folding factors including FkpA, SurA, Skp, and DegP. Several lipoproteins and integral Omps were also identified, including members of the beta-barrel assembly machinery BamA-D. Due to the overexpression of Omps and the associated upregulation of the sigma E stress response, this occurrence of proteins involved in the biogenesis of OMPs was expected^36^.

**Table 1:** Bacterial strains and plasmids used in this study and their source

Strain	Source
BL21(DE3)	Sigma Aldrich
BL21(DE3)*omp8*	Prilipov et al^30^.
pY03	Thoma et al^46^.
pY27	This study
pY161	This study
pYOX	This study
pY191	This study

### OMVs show densely packed Omps

**Fig. 2:**
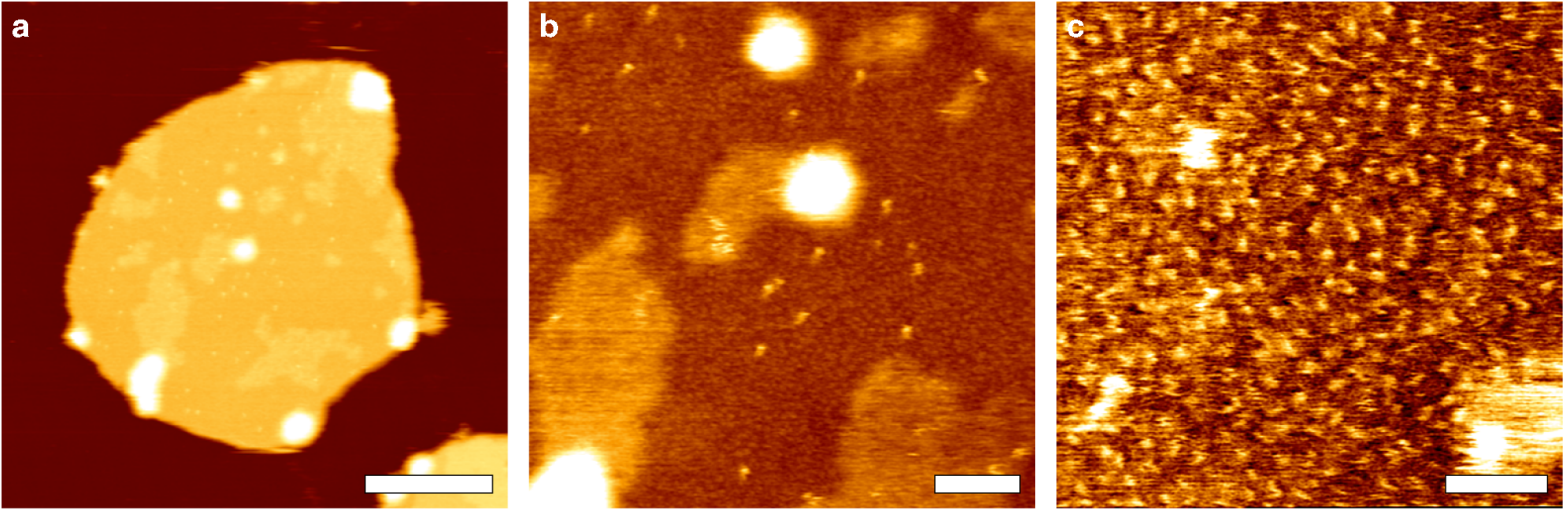
High-resolution AFM imaging of OMVs collected from *E. coli* overexpressing Omps. **a** Overview AFM topograph of an OMV enriched in Tsx adsorbed to mica. Upon adsorption to mica the OMVs opened as single layered membrane patches. For topographs of other OMV types see Supplementary Fig. 4. **b** Intermediate resolution topograph of the surface of the OMV shown in (**a**). Unstructured areas (lighter) alternate with homogeneously structured areas (darker). Both areas contain sparsely distributed particles protruding higher from the membrane. **c** High-resolution topograph of a lower protruding area reveals Omps (single protrusions) in densely packed arrangements. AFM topographs were recorded using contact-mode AFM in buffer solution (DPBSS) at room temperature (see Methods). The full color ranges of the topographs correspond to vertical scales of **a** 16 nm, **b** 4 nm and **c** 1 nm. Scale bars, 200 nm (**a**), 50 nm (**b**) and 20 nm (**c**)

To examine the membranes of OMVs containing overexpressed Omps visually, the vesicles were adsorbed to mica and imaged by AFM (Fig. 2 and Supplementary Fig. 4). The AFM topographs showed membrane patches ranging from 50 to 500 nm in diameter and 7 to 9 nm in height (Supplementary Fig. 5). The topographs thus suggested that the OMVs opened upon adsorption to the mica surface, resulting in single layered membranes. Single membranes of OMVs enriched in BamA were slightly higher (8.8 ± 0.4 nm, mean ± SD) than OMVs enriched in FhuA (7.9 ± 0.3 nm), OmpG (6.9 ± 0.3 nm), and Tsx (6.8 ± 0.3 nm), which correlates with the height differences of these Omps found in crystal structures^37–40^. In comparison, OMVs prepared from BL21(DE3) not overexpressing any Omp showed heights of 8.1 ± 0.5 nm. Despite these differences in height, all OMV preparations showed similar morphologies. The observed diameter of membrane patches corresponded to OMVs having a diameter between 25 and 250 nm, a size range that matches the vesicles observed by TEM. High-resolution topographs showed distinct domains within the adsorbed membrane patches, which differed a few Ångström in height by which they protruded from the lipid membrane (e.g., ≈0.3 nm for OMVs containing overexpressed Tsx) (Fig. 2a). We could not reveal high-resolution AFM topographs of the higher protruding OMV domains (Fig. 2b), most probably because these domains were structurally flexible. However, high-resolution AFM of the lower protruding domains showed individual Omps, which were densely packed (Fig. 2b, c). Most Omps observed in these lower protruding OMV domains appeared homogeneous in height with a few sparsely distributed Omps appearing higher. The latter Omps most probably represented large residual Omps such as FhuA or BamA, which remained left in the OmpA-, OmpF-, OmpC-, and LamB-depleted outer membrane of the *E. coli* strain used. From such high-resolution topographs, we concluded that the membranes of OMVs contained Omps in densely packed arrangements.

### OmpG in OMVs shows native fold

Our analysis suggested that OMVs can be prepared from *E. coli* BL21(DE3)omp8 to contain the overexpressed Omp at high density. To determine whether the overexpressed Omp was folded correctly in the membranes of OMVs we applied AFM-based single-molecule force spectroscopy (SMFS)^41–43^. SMFS pushes the tip of the AFM cantilever onto a membrane protein to non-specifically attach a terminal end^44^. Retraction of the cantilever builds up a mechanical force that stretches the terminal end and unfolds and extracts the protein from the membrane^44^. During this unfolding and extraction process, the deflection of the cantilever and the tip-membrane distance is recorded in a so-called force-distance curve. Thereby, the individual force peaks of the force-distance curve record the stepwise unfolding and extraction of the membrane protein. This saw-tooth-like unfolding pattern recorded is highly reproducible and unique for the structure and functional state of the membrane protein^41,43^. SMFS can therefore be used to simultaneously identify a protein and to assess its folded and misfolded state^45–47^.

**Fig. 3:**
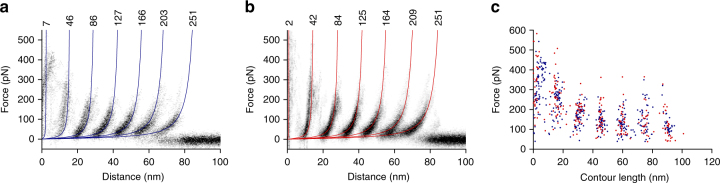
Unfolding pattern confirms the native fold of OmpG in OMVs. **a** Density map of 46 superimposed force-distance curves recorded from OMVs collected from *E. coli* overexpressing OmpG. **b** Density map of 33 superimposed force-distance curves recorded of OmpG reconstituted in lipid bilayers assembled from *E. coli* polar lipid extract. Blue (**a**) and red (**b**) curves are worm-like chain (WLC) curves indicating the mean contour length of each unfolding force peak. Contour lengths are given in number of amino acids above the WLC curves. **c** Force vs. contour length plot showing the fitting results of every detected force peak in all force-distance curves. Blue dots are from the measurements on OMVs shown in (**a**), red dots are from measurements on reconstituted OmpG shown in (**b**). Each dot represents the analysis of one unfolding force peak. SMFS was recorded in buffer solution (DPBSS) at room temperature (see Methods)

For SMFS we adsorbed OMVs released and purified from *E. coli* BL21(DE3)omp8 overexpressing OmpG to mica and located membrane patches by AFM imaging. We then repeatedly pushed the AFM tip onto the membrane surface in order to facilitate the non-specific attachment of an OmpG^44^. If the OmpG attached to the AFM tip, the subsequent retraction of the tip could induce the mechanical unfolding of the membrane protein. For each approach and retraction cycle a force-distance curve was recorded. In about 0.3‰ of attempts (*n* = 162,657), the force-distance curves showed the distinct saw-tooth like unfolding pattern of an Omp^46,48,49^. This indicated that the OmpG density in OMVs was sufficient to pick up single OmpG molecules for SMFS. The majority (>95%) of these force-distance curves showed an unfolding pattern with seven predominant force peaks (Fig. 3a). The unfolding pattern showed a high similarity to that recorded upon mechanically unfolding reconstituted OmpG into lipid bilayers assembled from *E. coli* polar lipid extract (Fig. 3b). It was previously shown, that each force peak of this unfolding pattern describes an unfolding step and intermediate of OmpG^46,48,49^. It was also shown, that this pattern is specific for pulling the N-terminal end of OmpG, which faces the periplasmic space of *E. coli*^46,48,49^. The OMV membranes thus exposed their periplasmic surface to the buffer solution. Fitting the position of each force peak in every force-distance curve with the worm-like chain (WLC) model^50^ revealed the contour lengths OmpG unfolded in each unfolding step. The seven force peaks indicated seven unfolding steps, which showed very similar contour lengths, independent whether OmpG was unfolded from the native membrane of OMVs or from reconstituted samples (Fig. 3c). The deviation of the force peak positions detected among both samples was less than the accuracy of the measurement^48^. Furthermore, the forces required to induce the stepwise unfolding of OmpG were similar for both samples, indicating that OmpG embedded in the native outer membrane and OmpG reconstituted into membranes of *E. coli* polar lipid extract show similar mechanical stability. In summary, we can conclude that the OmpGs unfolded from OMVs were folded in their native structure.

### FhuA in OMVs unfolds differently from reconstituted FhuA

**Fig. 4:**
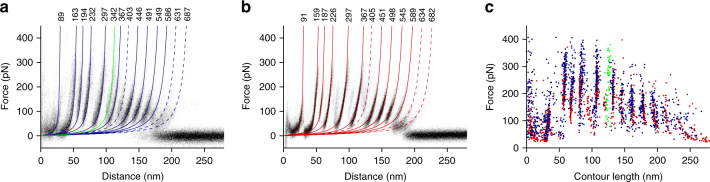
Unfolding pattern of the Omp FhuA depends on membrane environment. **a** Density map of 82 superimposed force–distance curves recorded from OMVs containing overexpressed FhuA. **b** Density map of 80 superimposed force–distance curves recorded from FhuA, reconstituted in lipid bilayers assembled from *E. coli* polar lipid extract. **c** Force vs. contour length plot showing the result obtained from fitting every force peak of every force–distance curve superimposed (**a**, **b**). Blue and green dots are from measurements from OMVs, red dots are from measurements from reconstituted FhuA. Colored curves (**a**, **b**) are WLC curves indicating the mean contour length of each unfolding force peak. Dashed and solid WLC curves indicate force peaks present in less than and more than 30% of the superimposed force-distance curves, respectively. Contour lengths are given in number of amino acids above the WLC curves. SMFS was recorded in buffer solution (DPBSS) at room temperature (see Methods)

Next, we repeated SMFS with OMVs containing high levels of FhuA. As for OmpG, ≈0.3‰ of 280528 force-distance curves recorded in the approach and retraction cycles showed a force peak pattern that resembled a saw-tooth like unfolding pattern typically found for Omps^46,48,49,51^. The majority (>90%) of these force-distance curves showed 12 predominant force peaks (Fig. 4a), which was similar to the unfolding pattern previously recorded upon mechanically unfolding of FhuA reconstituted into *E. coli* polar lipid bilayers (Fig. 4b)^46^. It has been shown, that this pattern is specific for mechanically pulling the periplasmic N-terminal plug domain of FhuA^46^. The SMFS experiment thus confirmed that also these OMV membranes exposed their periplasmic surface to the buffer solution. Fitting every individual force peak in every force-distance curve with the WLC model^50^ revealed that most of the force peaks occurred at the same contour lengths in both FhuA samples (Fig. 4c). However, the unfolding pattern of FhuA from OMVs showed a prominent force peak at contour length ≈342 amino acids (Fig. 4a). This force peak was rarely observed upon mechanically unfolding of FhuA reconstituted into lipid bilayers (Fig. 4b)^46^. Analysis of the individual force-distance curves recorded upon unfolding FhuA from OMVs showed that the force-distance curves could be divided into two distinct classes (Supplementary Fig. 6). The first class contained the majority of the force-distance curves (*n* = 51/82), which showed the same unfolding pattern recorded for FhuA reconstituted into lipid bilayers. The force-distance curves of the second class (*n* = 31/82) showed no force peak at ≈367 amino acids, but contained a force peak at ≈343 amino acids instead. No force-distance curve was found that contained both force peaks in this region. In contrast, all force-distance curves recorded from FhuA reconstituted into lipid bilayers showed the force peak at ≈367 amino acids. This local alteration in the unfolding pattern indicated that in the native membrane FhuA can unfold via two alternative pathways.

To determine the origin for this different unfolding behavior we assigned the structural segments unfolded in each unfolding step to the secondary structure of FhuA (Supplementary Fig. 6b, d). The unfolding pathway given by the first class of force-distance curves described the stepwise unfolding of individual beta-hairpins, except for the N-terminal plug domain of FhuA^46^. The unfolding pathway given by the second class of force-distance curves also described the stepwise unfolding of individual beta-hairpins, except for beta-hairpins 4 and 5 (Supplementary Fig. 6e, f). These two beta-hairpins 4 and 5 unfolded first a single beta-strand and then three beta-strands. FhuA has previously been shown to interact with LPS via an LPS-binding site^52^. This site is located in the transmembrane region of beta-hairpins 4 and 5 (Supplementary Fig. 7), which coincides with the location of the altered unfolding pattern of FhuA in OMVs. Because the presence of LPS is the main difference between reconstituted FhuA samples and FhuA-enriched OMVs, it may be speculated that the locally altered unfolding behavior stems from interactions of FhuA with LPS.

### Tsx and BamA show hairpin-wise unfolding

**Fig. 5:**
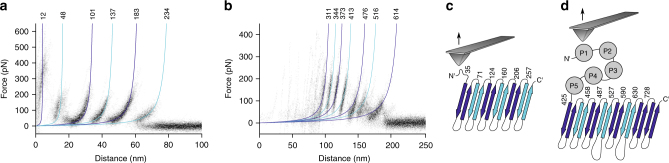
Unfolding pattern of the Omps Tsx and BamA recorded by SMFS. **a** Density map of 63 superimposed force-distance curves recorded from OMVs collected from *E. coli* overexpressing Tsx. **b** Density map of 73 superimposed force-distance curves recorded from OMVs collected from *E. coli* overexpressing BamA. Colored curves (**a**, **b**) are WLC curves indicating the mean contour length of each unfolding force peak. Contour lengths are given in number of amino acids above the WLC curves. **c** Secondary structure cartoon of Tsx with the structural segments unfolding in steps equally colored. A contour length of 23 amino acids was added to the contour length of every force peak to compensate the shift in distance, which can result from picking up Tsx at different positions along the N-terminus with the AFM tip. **d** Secondary structure cartoon of BamA with the structural segments unfolding in steps equally colored. A contour length of 114 amino acids was added to the contour length of every force peak to compensate the shift in distance resulting from picking up BamA at different positions along the POTRA domains with the AFM tip. To account for the disulfide bridge C690-C700 of BamA, we subtracted 9 amino acids to assign the unfolding steps exceeding a contour length of 690 amino acids to the secondary structure. The contour length of each unfolding force peak/unfolding step (**c**, **d**) is given in number of amino acids. SMFS was recorded in buffer solution (DPBSS) at room temperature (see Methods)

SMFS of OmpG and FhuA showed their native folds in OMVs. We thus expanded our experiments to characterize the mechanically induced unfolding of two additional Omps: the nucleotide-specific channel Tsx, a 12-stranded transmembrane beta-barrel, and the outer membrane assembly factor BamA, a two-domain protein comprising a 16-stranded transmembrane beta-barrel domain and a soluble periplasmic domain, consisting of five POTRA repeats (Fig. 5). Both membrane proteins have not yet been studied by SMFS. About 0.6‰ of 103‘038 force-distance curves recorded from OMVs containing overexpressed Tsx recorded a pattern of six reoccurring unfolding force peaks (Fig. 5a). Fitting of each force peak in every force–distance curve with the WLC model obtained their mean contour lengths of 12, 48, 101, 137, 183, and 234 amino acids (Fig. 5a). Locating the six unfolding force peaks in the secondary structure of Tsx revealed that each unfolding step is shaped by one of the six beta-hairpins of the protein (Fig. 5c). This result is consistent with the hairpin-wise unfolding behavior previously observed for other OMPs such as OmpG and FhuA^46,48^. We therefore conclude that Tsx was folded correctly in OMVs. Finally, we mechanically unfolded the core protein of the Bam complex, BamA, from OMVs. About 0.3‰ of 258‘760 force–distance curves recorded from OMVs containing overexpressed BamA showed an unfolding pattern that varied considerably in length ranging from 100 to 220 nm. All of these force–distance curves showed a pattern of seven force peaks at their end whereas the force peaks distributed irregularly at the beginning of the curves. Alignment of the force–distance curves to compensate for their relative distance shift showed the reproducibility of these seven force peaks (Fig. 5b). The observed length-variability is characteristic for membrane proteins having a large soluble domain and to which the AFM tip can attach non-specifically at multiple interaction sites^46,49^. Fitting of the seven re-occurring force peaks with the WLC model revealed that they were located at contour lengths of 311, 344, 373, 413, 476, 516, and 614 amino acids. We thus speculated that the disordered region at the beginning of the force–distance curves resulted from the unfolding the five POTRA domains and that the seven force peaks describe the stepwise unfolding of the beta-barrel of BamA. To compensate the shift resulting from picking up BamA at different positions along the POTRA domains, we added a contour length of 114 amino acids to the contour length of every force peak and assigned the structural segments by which BamA unfolded stepwise. The force peaks could then be assigned to the unfolding of the eight beta-hairpins forming the beta-barrel of BamA, with beta-hairpins 7 and 8 unfolding together (Fig. 5d). In this scenario, besides the last two beta-hairpins, also BamA showed the typical hairpin-wise unfolding behavior of a transmembrane beta-barrel protein in response to mechanical pulling forces.

## Discussion

Our experiments illustrate the suitability of OMVs as a platform for the overexpression and biophysical characterization of selected Omps. Overexpression was achieved by using *E. coli* BL21(DE3)omp8 that lacks all major Omps, which shifted the protein content of the OMVs in favor of the overexpressed Omp. In the membrane of the OMVs the overexpressed Omp was present at high density and sufficient purity. Our experiments showed that the fraction of overexpressed Omp over background proteins is sufficient to perform single molecule measurements and that these Omps were correctly inserted and folded into the OMV membrane. However, a basal content of other proteins was always present in OMVs. Many of these basal proteins are considered to be involved in Omp biogenesis. The essential nature of this processes suggests that this protein background cannot be circumvented. Nevertheless, the presence of additional proteins is one of the features that characterizes a native membrane environment. The reduced purity compared to reconstituted samples therefore is a tolerable tradeoff, compared to the benefit of a truly native membrane environment. This includes the native lipid composition, the lipid membrane asymmetry, and the controlled orientation of membrane proteins, three features, which cannot be accomplished by any reconstitution method. It has been previously shown, that the structure and function of membrane proteins, including Omps, can depend sensitively on the lipid composition, asymmetry and the direction of their insertion into the membrane^4–14^. Such problems are avoided by investigating membrane proteins in their native membrane environment. Furthermore, the use of *E. coli* as an overexpression platform of Omps, which are secreted in OMVs comes with the practical advantage of short preparation times. In contrast to conventional Omp purification and reconstitution approaches, which can require days up to weeks, the preparation of OMVs is a matter of hours. We expect that these considerable advantages of preparing Omps in the native outer membrane of OMVs will have potential impact on their structural and functional characterization by biophysical methods other than those applied in our study. In particular studying Omps in the native membrane environment might prove beneficial for studying the effectiveness of antibiotics and their interactions with Omps and the outer membrane, as the sensitivity of bacteria to antibiotics is directly influenced by the lipid and protein composition of the outer membrane^53,54^.

In our experiments, we did not observe any difference between the unfolding pattern of reconstituted OmpG and OmpG in OMVs. However, the unfolding pattern of FhuA was altered by new unfolding intermediates formed in the structural region involved in binding the LPS of OMVs. In fact, the functional state of several Omps such as omptin proteases has been reported to be sensitive to the presence of LPS^55,56^. Thus, using OMVs as platform to study Omps requiring the presence of LPS appears appealing. In particular, the presence of LPS in OMVs can be useful for AFM-based single-molecule studies. It has been reported that after adsorption of reconstituted proteoliposomes the exposed loop regions of membrane proteins can interact with the mica support, which restricts their mobility in the membrane^57^. This effect can be minimized by using membranes freely spanning across holes or by introducing spacer molecules that separate the membrane from the support^58^. When using OMVs the sugar-moieties of LPS act as a natural spacer to minimize the interactions between Omps and support. Even the shortened LPS expressed in BL21 strains, which only contains a truncated core oligosaccharide but lacks O-antigen^59,60^, would constitute a ≈2 nm long spacer. Such distance is in good agreement with the membrane height measured here of OMVs and which corresponds to a membrane thickness of ≈7–8 nm. This thickness is higher than that typically observed for proteoliposome membranes ≈5 nm directly adsorbed onto the supporting mica^61^. If needed, using smooth type LPS expressing *E. coli* strains could provide much longer spacers of several nm.

Being specialized in AFM we here focused on the use of OMVs for AFM-based approaches. However, we foresee that it is of great interest to study Omps in their native membrane environment by other methodologies as well^26^. With a protein-purity of up to 90%, OMVs might be used in cryo-electron microscopy or solid-state NMR spectroscopy for structural studies of Omps. However, OMVs may also be used for functional studies of Omps in assays, which so far relied on reconstituted proteoliposomes.

## Methods

### Cloning

See Table 1 for bacterial strains and plasmids. The ompG gene was amplified from MG1655 genomic DNA in 2 fragments using the primers 1 and 2, as well as primers 3 and 4 and sequentially subcloned into a pET21a plasmid between the NdeI and NcoI, and NcoI and HindIII sites respectively, resulting in pY27. The bamA gene was amplified from MG1655 genomic DNA using primers 5 and 6 and subcloned into a pET21a vector between the NdeI and SacI sites, resulting in pY161. For further expression of outer membrane proteins (Omps) we created plasmid pYOX, a pET21 plasmid in which the sequence 5′-AAA AAG TTA TTA CCC TGT ACC GCA CTG GTG ATG TGT GCG GGA ATG GCC TGC GCA CAG GCC ATG GGC AGC AGC CAT CAT CAT CAT CAT CAC AGC AGC GGC GAA AAC CTG TAC TTC CAG-3′ was inserted between the NdeI and BamHI restriction sites. This sequence encodes the periplasmic export signal peptide of the ompG gene, followed by a short peptide linker, a His_6_-tag and a TEV protease cleavage site and results in an N-terminal elongation of 22 amino acids for Omps cloned into this plasmid, which can act as a “handle” to facilitate the interaction with the AFM stylus in single-molecule force spectroscopy experiments. The tsx gene was amplified from MG1655 genomic DNA using primers 7 and 8 and subcloned into the pYOX plasmid between the BamHI and HindIII restriction sites, resulting in pY191. Primer sequences are given in Supplementary Table 2.

### Outer membrane vesicle production and purification

Bacterial cultures were inoculated at a ratio of 1/100 from overnight cultures and grown in 250 mL LB medium (Difco), supplemented with 100 µg mL^–1^ ampicillin (Sigma Aldrich) in baffled 1 L Erlenmeyer flasks under vigorous shaking at 37 °C. When cultures reached an optical density of OD_600_ ≈ 0.5 protein expression was induced by addition of 0.5 mM isopropyl-β-d-thiogalactoside (IPTG, Sigma Aldrich). When the optical density reached OD_600_ ≈ 0.95 bacteria were removed by centrifugation at 10,000x*g* for 10 min. The supernatant was filtered through 0.45 µm filter units (Merck Millipore). OMVs were collected by centrifugation (38,400x*g* for 2 h), resuspended in 25 mL Dulbeccos phosphate buffered saline with added magnesium and calcium (DPBSS, Sigma Aldrich), and collected by ultracentrifugation (100,000x*g* for 1 h). OMVs were resuspended in a final volume of 250 µL DPBSS and stored at –80 °C.

### Reconstitution of outer membrane proteins

Proteoliposomes containing reconstituted Omps were prepared as described for FhuA^46^. Briefly, to express Omps under leaky expression conditions in BL21(DE3)omp8 bacterial cultures were inoculated at a ratio of 1:100 from overnight cultures and grown in 2 L LB medium (Difco), supplemented with 100 µg mL^–1^ ampicillin (Sigma Aldrich) in 5 L Erlenmeyer flasks at 20 °C for 24 h. Cells were harvested (5000x*g* for 12 min), resuspended in buffer (20 mM Tris-HCl, 100 mM NaCl, pH 8) and broken by sonication. Cell envelopes were collected by centrifugation (100,000 xg for 1 h) and inner membranes were solubilized in buffer (20 mM Tris-HCl, 2% (w v^–1^) N-lauroylsarcosine (Sigma Aldrich), pH 8). Outer membranes were collected by centrifugation (100,000 xg for 1 h) and solubilized in buffer (20 mM Tris-HCl, 150 mM NaCl, 1% (w v^–1^) n-dodecyl-*N,N*-dimethylamine-N-oxide (LDAO, Anatrace), pH 8). Omps were then bound to Protino Ni-NTA agarose (Macherey-Nagel), washed with buffer (20 mM Tris-HCl, 150 mM NaCl, 0.5% (w v^–1^) LDAO, pH 8) and buffer (20 mM Tris-HCl, 150 mM NaCl, 0.1% (w v^–1^) LDAO, 10 mM imidazole (Sigma Aldrich), pH 8), and eluted with buffer (20 mM Tris-HCl, 150 mM NaCl, 0.1% (w v^–1^) LDAO, 500 mM imidazole, pH 8). Elution buffer was exchanged to buffer (20 mM Tris-HCl, 150 mM NaCl, 0.1% (w v^–1^) LDAO, pH 8) using PD-10 desalting columns (GE Healthcare) and the protein concentration was adjusted to 1 mg mL^–1^. Omps were reconstituted into *E. coli* polar lipid extract (Avanti polar Lipids) at a lipid to protein ratio of 0.5 (w w^–1^) by dialysis driven detergent removal against buffer (20 mM Tris-HCl, 150 mM NaCl, 0.01% (w v^–1^) NaN_3_, pH 8) for 5 days at 30 °C with daily buffer exchange. Samples were stored at –80 °C.

### Transmission electron microscopy (TEM)

For cryo-transmission electron microscopy (TEM), 2.5 µL of OMV samples were applied to a glow-discharged (10 mA for 120 s) holey carbon grid (Quantfoil, Cu R2/1). The grid was blotted on both sides for 4 s in a FEI Vitrobot at 100% humidity and 4 °C, and frozen rapidly by plunging into liquid ethane. Images were collected at liquid nitrogen temperature using an electron microscope (Tecnai F20, FEI) operated at 200 kV and equipped with a direct electron detector (Falcon 3, FEI) at a magnification of 50,000× (step size of 2.08 Å per pixel at the specimen level).

### Atomic force microscopy (AFM) imaging

OMVs containing overexpressed Omps were adsorbed to freshly cleaved mica for 15 min in DPBSS at room temperature. The sample was gently rinsed with DPBSS to remove non-adsorbed membranes^62^. Then the sample was imaged using force–distance curve-based AFM (Nanoscope Multimode 8, Bruker) in DPBSS at room temperature in the PeakForce Tapping mode following a previously described protocol^63^. Briefly, the AFM was equipped with a 120 μm piezoelectric scanner (J scanner) and fluid cell. PEAKFORCE-HIRS-F-A cantilevers (Bruker) with a nominal spring constant of 0.35 N m^–1^, resonance frequency of 165 kHz in liquid and sharpened silicon tip with a nominal radius of ≈1 nm were used for imaging. Images were recorded at 2 kHz oscillation frequency, applying an imaging force of 100–150 pN, scanned at 1 line s^–1^, with a vertical oscillation amplitude of 15 nm and a resolution of 512 × 512 pixels. Image analysis was performed using the Nanoscope analysis software (version 1.5) and Gwyddion (version 2.48). High resolution AFM topographs of Tsx containing OMVs were recorded in contact mode AFM using a JPK NanoWizard II (JPK Instruments) and OMCL-RC800PSA cantilevers (Olympus) in DPBSS at room temperature. The contact force applied ranged from 150 to 250 pN and the scanning rate was 4 line s^–1^. Image analysis was performed using the using the AFM analysis software (JPK Data Processing software, version spm-5.9.52).

### Single-molecule force spectroscopy (SMFS)

Mechanical unfolding of Omps was performed as described^44^. Briefly, 1 µL of OMV solution was adsorbed to freshly cleaved mica supports in 75 µL DPBSS for 20 min at room temperature. Samples were washed with DPBSS several times. All AFM measurements were performed on a JPK NanoWizard II (JPK Instruments) using OMCL-RC800PSA cantilevers (Olympus). Cantilevers were calibrated using the thermal noise method^64^. Adsorbed OMVs were located by imaging in contact mode, followed by force spectroscopy measurements using a contact force of ≈750 pN for 0.5 s, followed by retraction at constant velocity of 2 µm s^–1^. SMFS experiments were performed over several days, using a new sample and new AFM cantilever every day

### Data analysis

Force–distance curves were pre-processed as described^65^. Briefly, force–distance curves were corrected for deflection sensitivity of the cantilever and coarse filtered for force peak patterns exceeding a length, which corresponds to ≈2/3 of the contour length of the fully unfolded and extended polypeptide. All force–distance curves that remained after filtering were analyzed using an automated approach^66^. For alignment force–distance curves were transformed to force vs contour length space using the worm-like chain (WLC) model with a fixed persistence length of 0.4 nm^67^ and binned with a contour length bin-size of 1 nm. The force–distance curve with the highest similarity to all other force–distance curves was identified based on the minimal Euclidean distance against all curves, and used as a template for alignment. After alignment all force peaks in every force–distance curve were identified by discrete-wavelet-transform-based noise reduction^68^ and fitted using the WLC model^69^. The resulting force/contour length value pairs were pooled and clusters of high point density were identified using the DBSCAN algorithm^70^ with the elliptical distance $\frac{{\Delta f2}}{{r_f2}} + \frac{{\Delta l2}}{{r_l2}} \le 1$, where ∆*f* is the force distance between two points and ∆*l* is the contour length distance between two points), force radius *r*_*f*_ of 50 pN, contour length radius *r*_*l*_ of 1 nm, and a core point threshold for clustering of 7 points. For every cluster the mean contour length and mean force were calculated. The code used to analyze SMFS datasets is available from the corresponding author on reasonable request.

### Data availability

Experimental data are available upon reasonable request.

## Electronic supplementary material

Supplementary Information(PDF 1208 kb)
