# Supplementary material for: Protein-enriched outer membrane vesicles as a native platform for outer membrane protein studies
Source: Commun Biol. 2018 Apr 5;1:23. doi: 10.1038/s42003-018-0027-5 (PMC6123736; doi:10.1038/s42003-018-0027-5)
Supplement: Supplementary file 1 — Supplementary Information(PDF 1208 kb) [file 42003_2018_27_MOESM1_ESM.pdf]

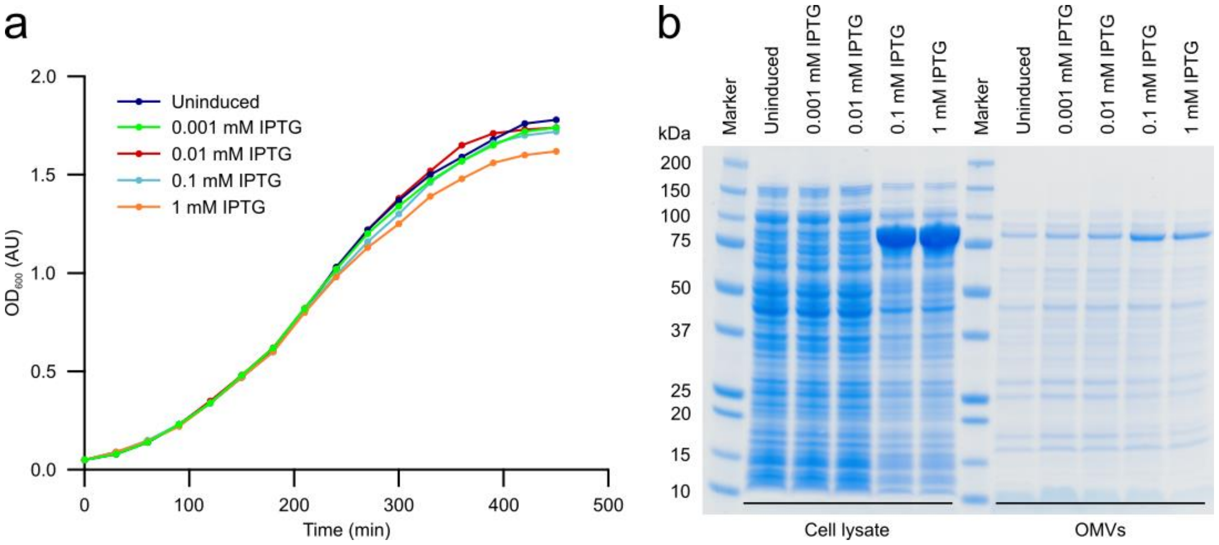

Supplementary Figure 1. Optimization of outer membrane protein (Omp) expression conditions. (**a**) Growth curves of BL21(DE3)omp8 expressing FhuA from plasmid pY03 at different induction levels. Bacterial cultures were grown in 50 ml LB medium supplemented with 100 µg ml^{-1} ampicillin in baffled 250 ml Erlenmeyer flasks under vigorous shaking at 37 ˚C. Expression of FhuA was induced when cultures reached an optical density of $OD_{600} \approx 0.4$ with IPTG (final concentrations given in the legend). To identify the optimal time point to stop bacterial growth and collect OMVs the infliction point of the growth curves was determined at $OD_{600} \approx 0.9$. As mass spectrometry of OMVs collected from bacteria cultures in the stationary phase showed high numbers of cytoplasmic and inner membrane proteins (not shown), for our studies we collected OMVs from cultures in the late exponential growth phase. (**b**) SDS-PAGE of cell lysates and OMVs collected after expression of FhuA in BL21(DE3)omp8 at different induction levels. Bacterial cultures were grown in 50 ml LB medium supplemented with 100 µg ml^{-1} ampicillin in baffled 250 ml Erlenmeyer flasks under vigorous shaking at 37 ˚C. Expression of FhuA was induced when cultures reached an optical density of $OD_{600} \approx 0.4$ with IPTG (final concentrations given in the figure). Bacterial growth was stopped when cultures reached an optical density of $OD_{600} \approx 0.95$ and OMVs were prepared as described (**Methods**). Cell pellets of cultures induced with IPTG concentrations < 0.1 mM were brown, whereas cell pellets of cultures induced with higher IPTG concentrations were white, indicating the formation of inclusion bodies. SDS-PAGE showed a prominent band at ~ 80 kDa in cell lysates from cultures induced with IPTG concentrations > 0.1 mM. SDS-PAGE of OMV preparations from the same cultures showed prominent bands at the same weight, the intensity of which increased with increasing IPTG concentrations. However, the intensity of the prominent band in OMVs did not increase further at IPTG concentrations between 0.1 and 1 mM indicating saturation of the expression levels.

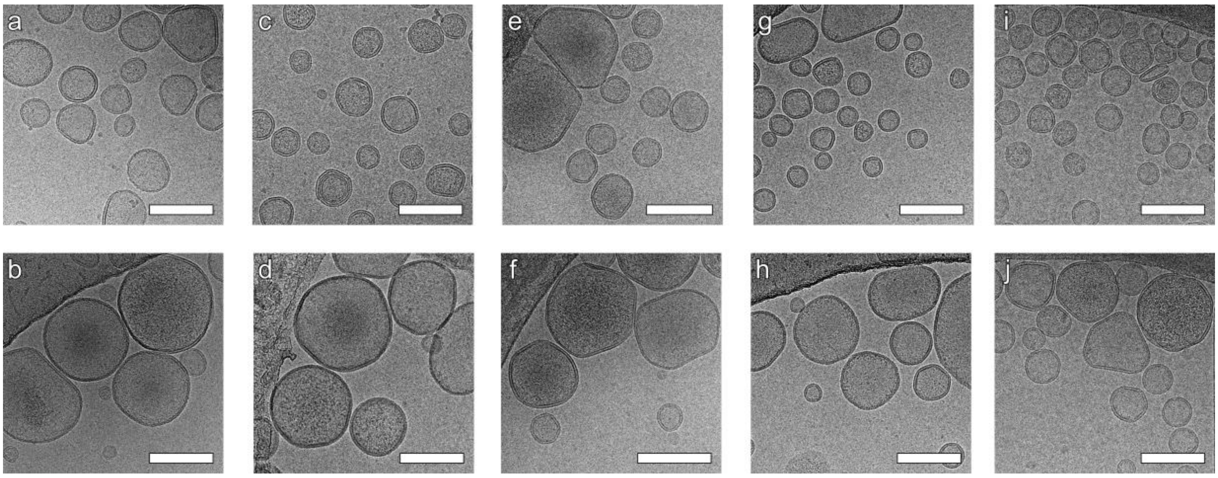

Supplementary Figure 2. Cryo-transmission electron microscopy (TEM) of OMVs. (**a,b**) Cryo-TEM images of OMVs collected from BL21(DE3) not overexpressing any Omp. (**c,d**) Cryo-TEM images of OMVs collected from BL21(DE3)omp8 overexpressing FhuA. (**e,f**) Cryo-TEM images of OMVs collected from BL21(DE3)omp8 overexpressing OmpG. (**g,h**) Cryo-TEM images of OMVs collected from BL21(DE3)omp8 overexpressing Tsx. (**i,j**) Cryo-TEM images of OMVs collected from BL21(DE3)omp8 overexpressing BamA. Scale bars, 150 nm.

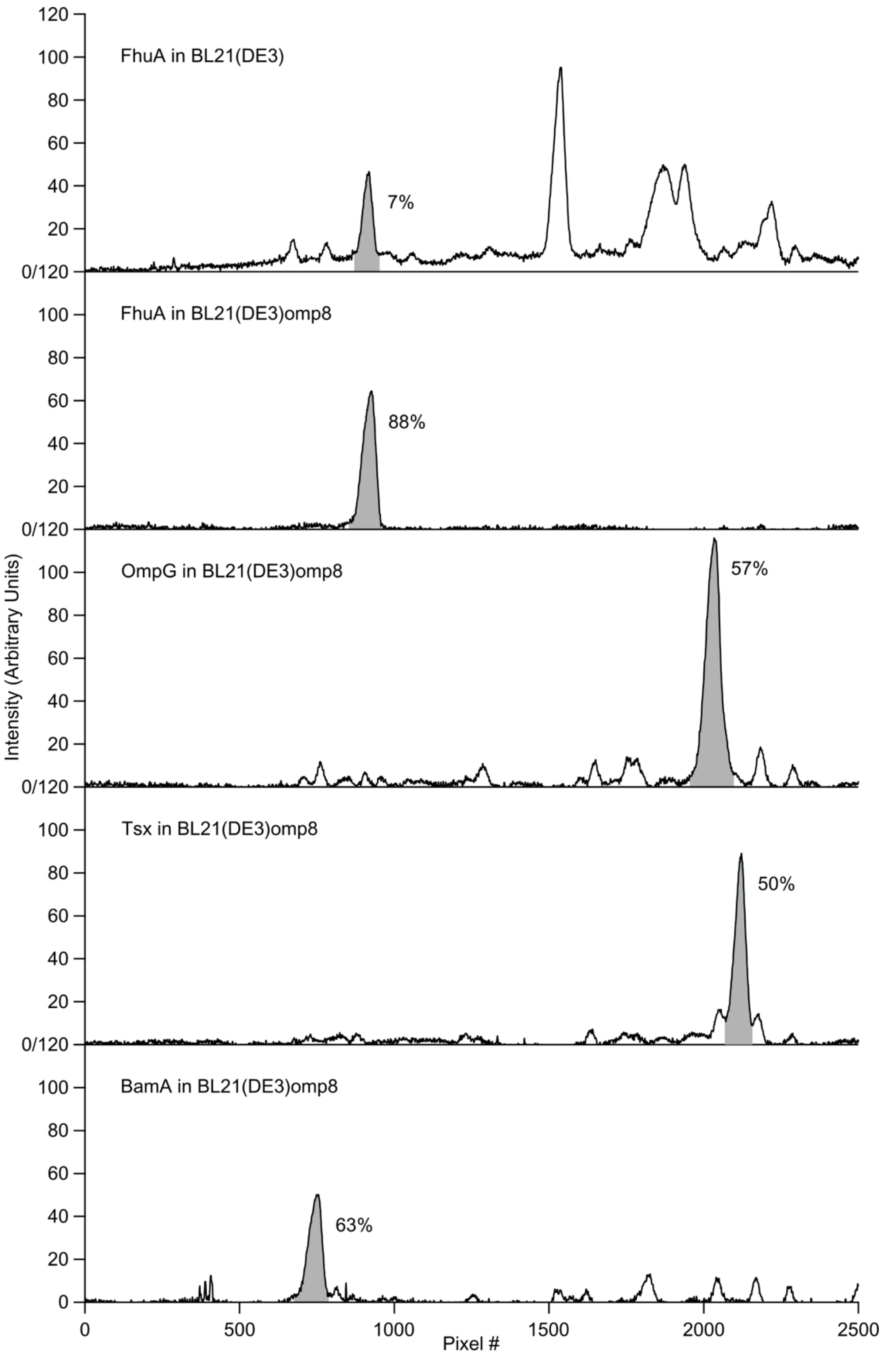

Supplementary Figure 3. Intensity profiles of SDS-PAGE gel from Figure 1c. Black lines represent the average of three individual line profiles extracted from each gel lane. The average of six line profiles extracted from empty regions between the gel lanes was taken as the baseline and subtracted from every profile. Grey areas are the selected peak areas. Numbers next to grey peaks give the fraction of the grey areas over the total area of the black line profiles (in percent). Line profiles were extracted using the software ImageJ (version 1.48v).

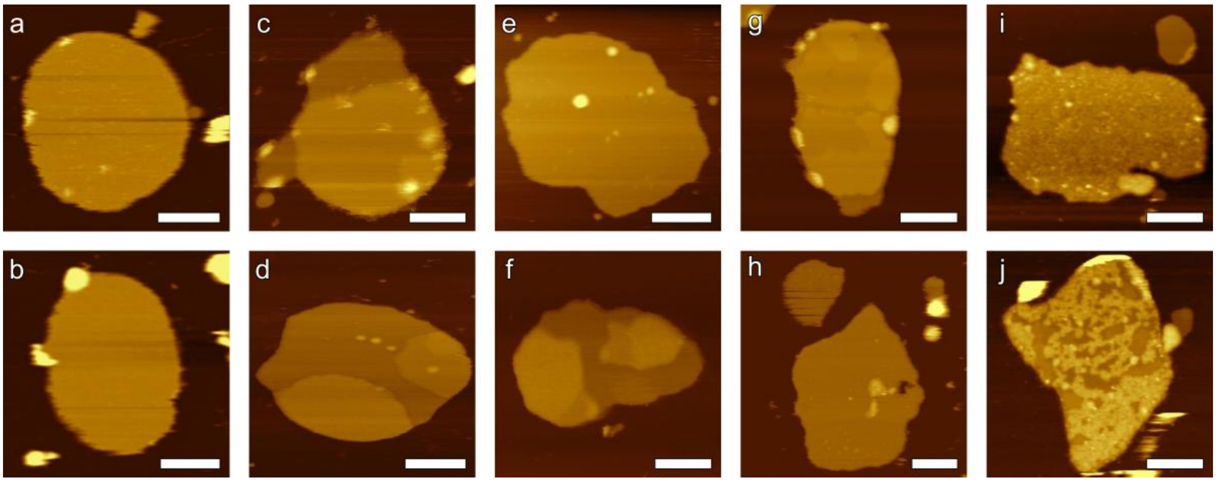

Supplementary Figure 4. AFM imaging of OMVs collected from *E. coli* overexpressing different Omps. Overview AFM topographs of OMVs collected from BL21(DE3) not overexpressing any Omp (**a**,**b**) and of OMVs collected from BL21(DE3)omp8 enriched in FhuA (**c**,**d**), OmpG (**e**,**f**), Tsx (**g**,**h**), and BamA (**i**,**j**) adsorbed to mica. Upon adsorption to mica the OMVs opened as single layered membrane patches. AFM topographs were recorded in buffer solution (DPBSS) at room temperature by force-distance curve based AFM (**c**-**f**,**i**,**j**) and contact mode AFM (**a**,**b**,**g**,**h**). AFM imaging was conducted in buffer solution as described (Methods). Scale bars. 150 nm (**c**), 200 nm (**a**,**b**,**d**,**e**,**g**,**h**), 100 nm (**f**), 300 nm (**i**) and 250 nm (**j**). The full color range of the topographs corresponds to a vertical scale of 21 nm.

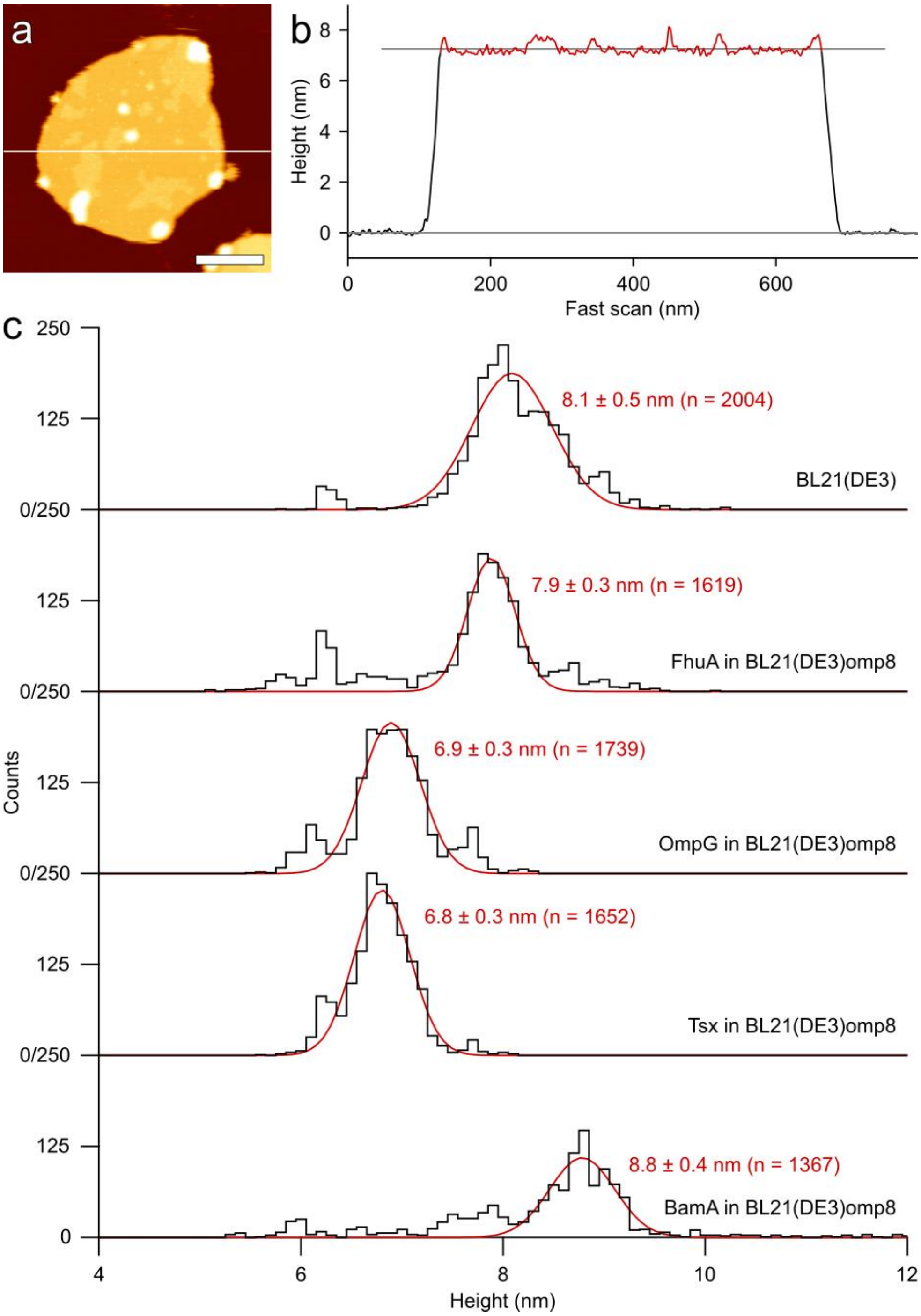

Supplementary Figure 5. Height of OMVs adsorbed to mica. (**a**) AFM topograph of an OMV adsorbed to mica. Upon adsorption to mica the vesicle burst open and thus appeared as single layered OMV membrane. The topograph was recorded in buffer solution (DPBSS) and at room temperature as described (**Methods**). Scale bar, 200 nm. The full color range of the topograph corresponds to a vertical scale of 16 nm. (**b**) Height profile extracted across the OMV membrane imaged in the AFM topograph (indicated by the white line in **a**). From such topographs the height of OMV membranes protruding from mica was measured (red line). (**c**) Histograms of height values recorded from each OMV preparation (black lines). For each OMV preparation the height values of 20 OMVs imaged by AFM were analyzed using the AFM analysis software (JPK data processing software, version spm-5.1.8). Distributions were fitted using a Gaussian function (red lines) to reveal mean $\pm$ SD height values. All membrane heights differed significantly from each other (one-way ANOVA test; $F(4,8376) = 1546.4$; MSE = 1.06×10^{15}; $p < 0.0001$).

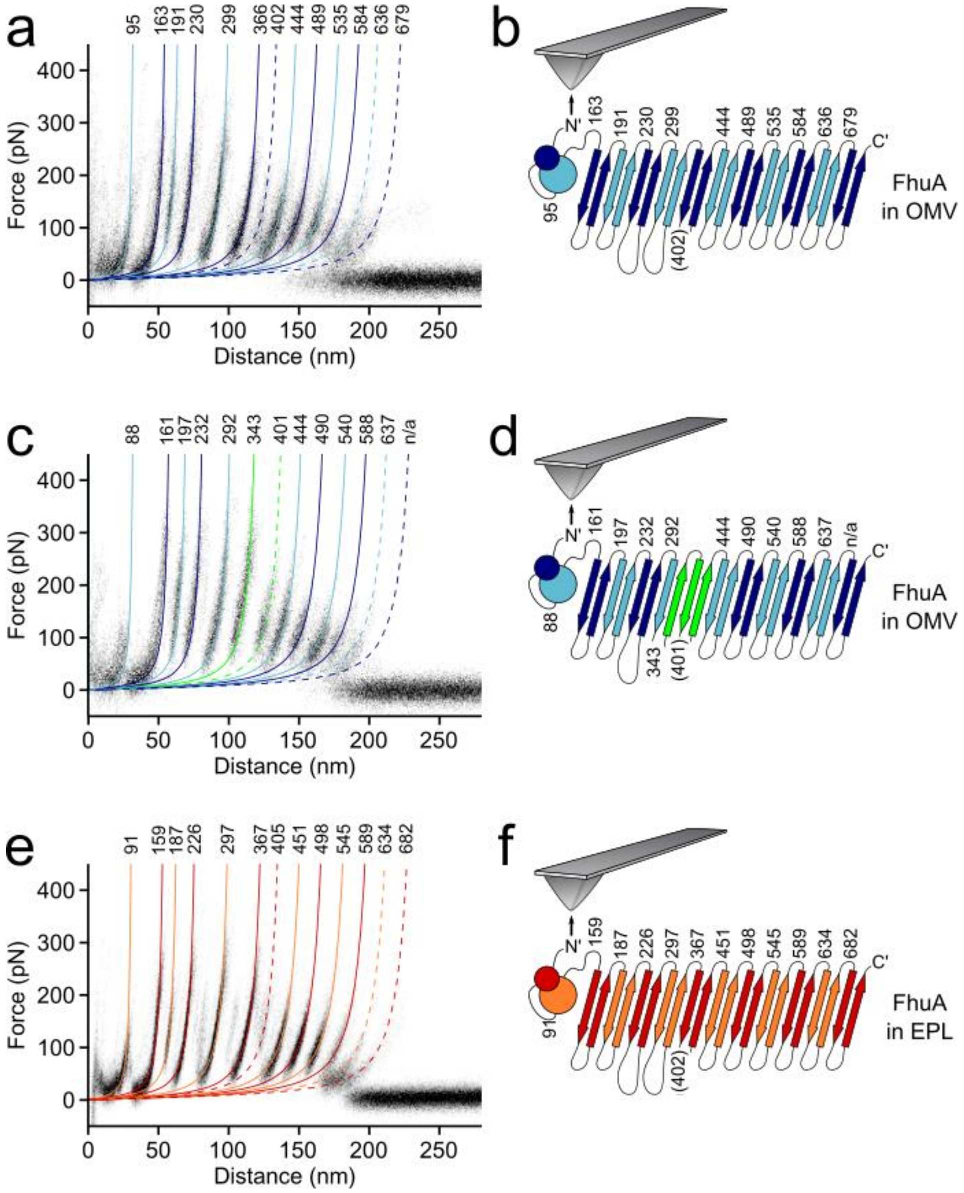

Supplementary Figure 6. FhuA shows different unfolding pathways depending on membrane environment. (**a,c**) FD curves recorded on FhuA from OMVs were separated into two distinct classes. (**a**) Density map of 51 superimposed FD curves of the first class of FD curves recorded upon unfolding FhuA from OMVs. Colored curves are worm-like chain (WLC) curves indicating the mean contour length of each unfolding force peak. Contour lengths are given in number of amino acids above the WLC curves. Data taken from **Figure 4a**. (**b**) Secondary structure cartoons of FhuA highlighting the structural segments unfolded in single steps detected in (**a**) (equally colored). Unfolding steps were located in the structure by subtracting the number of amino acids of the mean contour length of each unfolding step from the N-terminal end of FhuA. To account for the disulfide bridge C351-C362 of FhuA, we subtracted 10 aa to assign the unfolding steps exceeding a contour length of 351 aa to the secondary structure. (**c**) Density map of 31 superimposed FD curves of the second class of FD curves recorded upon unfolding FhuA from OMVs. (**d**) Secondary structure cartoons of FhuA highlighting the structural segments unfolded in single steps detected in (**c**) (equally colored). (**e**) Density map of 80 superimposed FD curves recorded from FhuA reconstituted in *E. coli* polar lipid extract. (**f**) Secondary structure cartoons of FhuA highlighting the structural segments unfolded in each single unfolding step described by a force peak in (**e**) (equally colored).

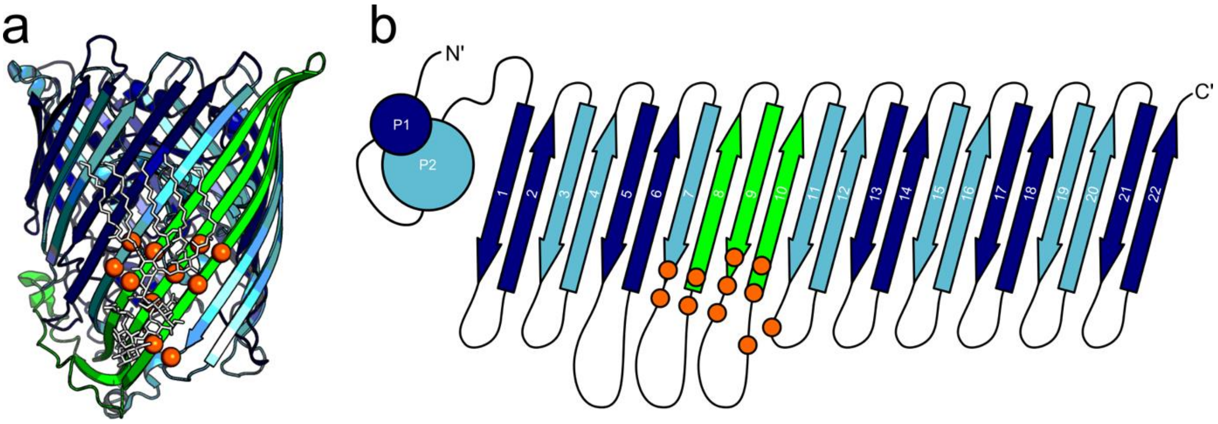

Supplementary Figure 7. Lipopolysaccharide (LPS) binding site of FhuA. (**a**) Tertiary structure cartoon of FhuA (blue and green, PDB-code 1QFG) with bound LPS (white). Orange spheres represent C-alpha atoms of residues involved in LPS binding. Beta-strands 8, 9, and 10 are highlighted in green. (**b**) Secondary structure cartoon of FhuA. Numbers (1-22) of all beta strands and structural segments of the plug domain (P1,2) are given in white. Orange circles indicate locations of residues involved in LPS binding. The periplasmic side of FhuA is facing upwards in both representations.

Subcellular location	Gene Name	Description	UniProt Accession
Cell Outer Membrane	bamA	Outer membrane protein assembly factor BamA OS=Escherichia coli O139:H28 (strain E24377A / ETEC) GN=bamA PE=3 SV=1	A7ZHR7
Cell Outer Membrane	bamB	Outer membrane protein assembly factor BamB [OS=Escherichia coli K-12]	P77774
Cell Outer Membrane	bamC	Outer membrane protein assembly factor BamC OS=Escherichia coli (strain K12) GN=bamC PE=1 SV=1	P0A903
Cell Outer Membrane	bamD	Outer membrane protein assembly factor BamD OS=Escherichia coli (strain K12) GN=bamD PE=1 SV=1	P0AC02
Cell Outer Membrane	emtA	Endo-type membrane-bound lytic murein transglycosylase A OS=Escherichia coli (strain K12 / DH10B) GN=emtA PE=3 SV=1	B1XAN4
Cell Outer Membrane	flgH	Flagellar L-ring protein OS=Escherichia coli (strain SE11) GN=flgH PE=3 SV=1	B6I9F5
Cell Outer Membrane	loiP	Metalloprotease LoiP [OS=Escherichia coli K-12]	P25894
Cell Outer Membrane	lpoA	Penicillin-binding protein activator LpoA [OS=Escherichia coli K-12]	P45464
Cell Outer Membrane	lpoB	Penicillin-binding protein activator LpoB OS=Escherichia coli (strain K12) GN=lpoB PE=1 SV=1	P0AB38
Cell Outer Membrane	mltA	Membrane-bound lytic murein transglycosylase A OS=Escherichia coli O157:H7 GN=mltA PE=3 SV=1	P0A936
Cell Outer Membrane	nlpE	Lipoprotein NlpE [OS=Escherichia coli K-12]	P40710
Cell Outer Membrane	pal	Peptidoglycan-associated lipoprotein OS=Escherichia coli (strain K12) GN=pal PE=1 SV=1	P0A912
Cell Outer Membrane	rcsF	Outer membrane lipoprotein RcsF OS=Escherichia coli O6:H1 (strain CFT073 / ATCC 700928 / UPEC) GN=rcsF PE=3 SV=1	P69412
Cell Outer Membrane	tsx	Nucleoside-specific channel-forming protein tsx OS=Escherichia coli (strain K12) GN=tsx PE=1 SV=1	P0A927
Cell Outer Membrane	yncE	Uncharacterized protein YncE [OS=Escherichia coli K-12]	P76116
Periplasm	agp	Glucose-1-phosphatase OS=Escherichia coli (strain K12) GN=agp PE=1 SV=1	P19926
Periplasm	ais	Lipopolysaccharide core heptose(II)-phosphate phosphatase OS=Escherichia coli (strain K12 / MC4100 / BW2952) GN=ais PE=3 SV=1	C4ZU94
Periplasm	bglX	Periplasmic beta-glucosidase [OS=Escherichia coli K-12]	P33363
Periplasm	chiA	Probable bifunctional chitinase/lysozyme OS=Escherichia coli (strain K12) GN=chiA PE=1 SV=2	P13656
Periplasm	cpdB	2',3'-cyclic-nucleotide 2'-phosphodiesterase/3'-nucleotidase [OS=Escherichia coli K-12]	P08331

Periplasm	cpoB	Cell division coordinator CpoB OS=Escherichia coli (strain K12) GN=cpoB PE=1 SV=2	P45955
Periplasm	cueE	Blue copper oxidase CueO [OS=Escherichia coli K-12]	P36649
Periplasm	cysP	Thiosulfate-binding protein OS=Escherichia coli (strain K12) GN=cysP PE=1 SV=1	P16700
Periplasm	dsbA	Thiol:disulfide interchange protein DsbA [OS=Escherichia coli K-12]	P0AEG4
Periplasm	dsbC	Thiol:disulfide interchange protein DsbC [OS=Escherichia coli K-12]	P0AEG6
Periplasm	efeB	Deferrochelatase/peroxidase EfeB [OS=Escherichia coli K-12]	P31545
Periplasm	efeO	Iron uptake system component EfeO [OS=Escherichia coli K-12]	P0AB24
Periplasm	fecB	Fe(3+) dicitrate-binding periplasmic protein [OS=Escherichia coli K-12]	P15028
Periplasm	fkpA	FKBP-type peptidyl-prolyl cis-trans isomerase FkpA OS=Escherichia coli (strain K12) GN=fkpA PE=1 SV=1	P45523
Periplasm	glnH	Glutamine-binding periplasmic protein OS=Escherichia coli (strain K12) GN=glnH PE=1 SV=1	P0AEQ3
Periplasm	glpQ	Glycerophosphoryl diester phosphodiesterase [OS=Escherichia coli K-12]	P09394
Periplasm	gltI	Glutamate/aspartate import solute-binding protein [OS=Escherichia coli K-12]	P37902
Periplasm	gsiB	Glutathione-binding protein gsiB [OS=Escherichia coli K-12]	P75797
Periplasm	hisJ	Histidine-binding periplasmic protein [OS=Escherichia coli K-12]	P0AEU0
Periplasm	lptA	Lipopolysaccharide export system protein lptA [OS=Escherichia coli K-12]	P0ADV1
Periplasm	lolA	Outer-membrane lipoprotein carrier protein [OS=Escherichia coli K-12]	P61316
Periplasm	mdoG	Glucans biosynthesis protein G [OS=Escherichia coli K-12]	P33136
Periplasm	mlaC	Probable phospholipid-binding protein mlaC [OS=Escherichia coli K-12]	P0ADV7
Periplasm	mppA	Periplasmic murein peptide-binding protein [OS=Escherichia coli K-12]	P77348
Periplasm	oppA	Periplasmic oligopeptide-binding protein [OS=Escherichia coli K-12]	P23843
Periplasm	osmY	osmotically-inducible protein Y [OS=Escherichia coli K-12]	P0AFH8
Periplasm	ppiA	Peptidyl-prolyl cis-trans isomerase A OS=Escherichia coli O157:H7 GN=ppiA PE=3 SV=1	P0AFL5
Periplasm	proX	Glycine betaine/proline betaine-binding periplasmic protein [OS=Escherichia coli K-12]	P0AFM2
Periplasm	ptrA	Protease 3 [OS=Escherichia coli K-12]	P05458
Periplasm	rsbB	Ribose import binding protein RbsB [OS=Escherichia coli K-12]	P02925

Periplasm	skp	Chaperone protein Skp OS=Escherichia coli O157:H7 GN=skp PE=3 SV=1	P0AEU9
Periplasm	surA	Chaperone SurA OS=Escherichia coli O6:H1 (strain CFT073 / ATCC 700928 / UPEC) GN=surA PE=3 SV=1	P0ABZ7
Periplasm	tolB	Protein TolB OS=Escherichia coli O6:K15:H31 (strain 536 / UPEC) GN=tolB PE=3 SV=1	Q0TJV4
Periplasm	ushA	protein UshA [OS=Escherichia coli K-12]	P07024
Periplasm	ybiS	Probable L,D-transpeptidase YbiS OS=Escherichia coli O6:H1 (strain CFT073 / ATCC 700928 / UPEC) GN=ybiS PE=3 SV=1	P0AAX9
Periplasm	yceI	Protein YceI [OS=Escherichia coli K-12]	P0A8X2
Periplasm	ygiW	Protein YgiW OS=Escherichia coli (strain K12) GN=ygiW PE=1 SV=1	P0ADU5
Periplasm	yhiJ	Protein yhjJ [OS=Escherichia coli K-12]	P37648
Periplasm	yraP	Uncharacterized protein YraP OS=Escherichia coli O157:H7 GN=yraP PE=3 SV=1	P64598
Cytoplasm	acpP	Acyl carrier protein OS=Escherichia coli O45:K1 (strain S88 / ExPEC) GN=acpP PE=3 SV=1	B7MJ81
Cytoplasm	tuf1	Elongation factor Tu 1 OS=Escherichia coli O1:K1 / APEC GN=tuf1 PE=3 SV=2	A1AGM6
Cell Inner Membrane/Cell Outer Membrane	yiaD	Probable lipoprotein YiaD OS=Escherichia coli (strain K12) GN=yiaD PE=1 SV=2	P37665
Cell Inner Membrane	nlpD	Murein hydrolase activator NlpD OS=Escherichia coli (strain K12) GN=nlpD PE=1 SV=1	P0ADA3
Cell Inner Membrane	prc	Tail-specific protease [OS=Escherichia coli K-12]	P23865
Cell Membrane	osmE	Osmotically-inducible lipoprotein E [OS=Escherichia coli K-12]	P0ADB1
Cell Membrane	rlpA	Rare lipoprotein A [OS=Escherichia coli K-12]	P10100
Cell Membrane	yajG	Uncharacterized lipoprotein YajG OS=Escherichia coli O6:H1 (strain CFT073 / ATCC 700928 / UPEC) GN=yajG PE=3 SV=1	P0ADA6
Cell Membrane	ybaY	Uncharacterized lipoprotein YbaY OS=Escherichia coli (strain K12) GN=ybaY PE=3 SV=1	P77717
Cell Membrane	ydcL	Uncharacterized lipoprotein YdcL [OS=Escherichia coli K-12]	P64451
Cell Membrane	yifL	Uncharacterized lipoprotein yifL [OS=Escherichia coli K-12]	P0ADN6
	bla	Beta-lactamase TEM OS=Escherichia coli GN=bla PE=1 SV=1	P62593
	ydgH	Protein ydgH [OS=Escherichia coli K-12]	P76177
	yjeI	Uncharacterized protein YjeI [OS=Escherichia coli K-12]	P0AF70

Supplementary Table 1. List of proteins identified by mass spectrometry. Only proteins that were identified in all samples prepared from BL21(DE3)omp8 are given. Subcellular location information was taken from the UniProt database. Proteins involved in protein folding are highlighted (grey).

Primer	Sequence
1	5’-GGG AAT TCC ATA TGA AAA AGT TAT TAC CC-3’
2	5’-CAG CCA TGG ATG GTG ATG GTG ATG ATG GGC CTG TGC GCA GGC CAT TCC-3’
3	5’-CAG CCA TGG GGA GGC TCT GGA GGC TCT GGA GAG GAA AGG AAC GAC TGG-3’
4	5’-CCC AAG CTT GAA CGA GTA ATT TAC G-3’
5	5’-ATA CAT ATG GCG ATG AAA AAG TTG CTC-3’
6	5’-AAA GAG CTC TTA CCA GGT TTT ACC GAT GTT AA-3’
7	5’-CAG GGA TCC GCT GAA AAC GAC AAA CCG C-3’
8	5’-CAA GCT TGT CAG AAG TTG TAA CCT ACT AC-3’

Supplementary Table 2. List of primers.
